# Where in the world is my tweet: Detecting irregular removal patterns on Twitter

**DOI:** 10.1371/journal.pone.0203104

**Published:** 2018-09-20

**Authors:** Joan C. Timoneda

**Affiliations:** Department of Government and Politics, University of Maryland, College Park, Maryland, United States of America; Cardiff University, UNITED KINGDOM

## Abstract

Twitter data are becoming an important part of modern political science research, but key aspects of the inner workings of Twitter streams as well as self-censorship on the platform require further research. A particularly important research agenda is to understand removal rates of politically charged tweets. In this article, I provide a strategy to understand removal rates on Twitter, particularly on politically charged topics. First, the technical properties of Twitter’s API that may distort the analyses of removal rates are tested. Results show that the forward stream does not capture every possible tweet –between 2 and 5 percent of tweets are lost on average, even when the volume of tweets is low and the firehose not needed. Second, data from Twitter’s streams are collected on contentious topics such as terrorism or political leaders and non-contentious topics such as types of food. The statistical technique used to detect uncommon removal rate patterns is multilevel analysis. Results show significant differences in the removal of tweets between different topic groups. This article provides the first systematic comparison of information loss and removal on Twitter as well as a strategy to collect valid removal samples of tweets.

## Introduction

Researchers across the social sciences are becoming increasingly interested in using Twitter data in their studies and in understanding its limitations [1–4]. There are good reasons for this interest. First, the data are extraordinarily abundant and readily available to the public through the company’s two main APIs (forward stream and backward search). This is particularly attractive in those social science fields in which data collection is often an arduous and expensive process. Second, the nature of the data can help address certain pressing questions on how social networks behave and how individuals interact with each other [5, 6].

A particularly important research agenda is to understand removal rates of politically charged tweets. The removal of tweets could take place for a number of different reasons, such as self-censorship, bot removal or active reporting. Yet, due to data limitations and/or lack of knowledge about how Twitter works, little research has sought to deal with this issue. This article provides strategies to understand removal rates on Twitter and to detect anomalies on politically charged topics. To this end, I first analyze the technical properties of Twitter’s APIs to understand the factors that may distort the analyses of removal rates. Data are collected on six different topics from both the search and streaming API for the same time interval and the samples are compared. Results show that the forward stream does not capture every possible tweet –between 2 and 5 percent of tweets are lost on average, even when the volume of tweets is low and the firehose not needed. Second, a multilevel model to detect uncommon removal rate patterns is used. I collect a separate dataset from Twitter’s streams on a total of 36 contentious and non-contentious topics. The multilevel results show significant differences in the removal of tweets between different topic groups. As expected, contentious tweets are removed at higher rates, with tweets related to groups such as the Islamic State deleted the fastest. However, somewhat surprisingly, non-contentious topics such as food or pets are also removed more frequently than tweets about important political figures, suggesting that users may curate these tweets more often than previously thought. This opens a new avenue of research into self-regulation and self-censorship on Twitter that reaches beyond politically polarizing issues. This article provides the first systematic comparison of information loss and removal on Twitter as well as a strategy to collect valid removal samples of tweets.

There are different ways to access Twitter data, but researchers usually tap into the company’s two Application Programming Interfaces, commonly known as the search or historical API and the streaming or dynamic API. The search API allows the public to access a temporary repository of tweets that includes a large sample of all tweets published during the week prior to the query. Older tweets are moved to Twitter’s permanent server, which is only accessible by request and is usually for pay. The streaming API, on the other hand, lets users capture tweets in real time. Programs such as Twarc or streamR set up ‘tracking’ streams that download all tweets that contain a particular keyword chosen by the researcher (Twarc is a Python-based program and streamR is R-based. Both also allow users to download timelines of particular Twitter users as well as access other forms of data. See https://github.com/DocNow/twarc for a description of Twarc). The difficulty with this stream is the impossibility of knowing ex-ante what interesting issues will become important in the future. Yet, researchers often have a good, if broad, idea of the topic of interest and can set up tracking streams with multiple keywords.

To get a better understanding of the data-generating process on Twitter, API performance is tested on three main fronts. First, I investigate how long it takes for the search API to update compared to the streaming API and at what point the convergence stops. This test determines whether the two streams share similar content or whether bias is prevalent on one stream or the other, following Gonzalez-Bailón et al. [7]. Second, the frequency with which tweets appear only in one of the two streams is explored. We would expect some terms to appear in the dynamic API but not in the search API, since the latter provides only a sample of tweets. Surprisingly, the opposite also occurs. Some tweets appear in the search stream but not in the forward stream, which is counterintuitive for those terms with low levels of activity. Third, I analyze how many tweets have been removed from Twitter and investigate whether it is more likely that a tweet has been removed from the search or streaming APIs. If we do not know the natural rate of removal of tweets for various topics, it is difficult to know the extent to which the two streams do not match.

This paper has been designed to appeal to researchers who want to use Twitter data on topics with medium and low levels of activity, and who want to know what is the best way to collect their data and what potential pitfalls exist with using either of the two streams Twitter offers. It also provides a simple template to test how the APIs work on any given topic. As regards removal rates, this is the first study that explores them in depth. The expected removal rate across a range of topics are shown at two different time points: within fifteen minutes and within a week of publication of the tweet. Taken together, the evidence shows that researchers should set up tweet collections using both the streaming and the search API.

## Materials and methods

Data were extracted directly from Twitter’s APIs. All the data from the streaming API were captured live, while the data from the search API were downloaded within one week of publication on Twitter. It is important to note that the streaming or dynamic API allows live access to all tweets as they become available. The only limitation is that the search term used cannot represent more than 1 percent of total Twitter traffic. The historic or search API provides a sample of the total volume of tweets produced within the previous 7 days that contain a certain keyword (all data have been accessed and stored in full compliance with Twitter’s terms of service).

### Data for time analysis

We know that tweets from the two APIs do not match perfectly [8, 9], but mismatches are usually attributed to the search stream producing a *sample* of past tweets that does not include all tweets present in the forward stream, which captures every tweet as it is published. Yet, we do not know exactly how and why this is so, or whether some tweets could indeed be in the search stream that are *not* in the forward stream. Additionally, mismatches could be attributed to waiting an insufficient amount of time between the publication of the tweet and trying to retrieve it from the forward or search streams. Indeed, it is plausible that the search stream may take longer to update than the forward stream. However, neither hypothesis has been tested, and it is important to understand how the streams update their information and the extent to which they match in order to properly analyze removal rates of tweets.

All of the samples of tweets collected in this paper followed the same methodology. In R, using the packages streamR and twitteR, I created the function collectTweets() that, for any given term or set of terms, collected tweets for the period of time specified from both APIs on a given set of terms (R version 3.1.3 was used. Python version 3.5.1 was used to run Twarc). The process generated a multidimensional list, which stored all the information about the tweets plus a set of markers for whether the tweet was in the dynamic API, appeared in the search API, or had been removed. To be certain that the tweet had in fact been removed, the url.exists() function from the RCurl package was used to check each tweet’s URL on Twitter’s website. If the function returned an error, the tweet was coded as having been removed.

To test whether the wait time between collections makes a difference to the likelihood that a tweet will be in the both APIs, the following wait intervals between forward and backward collection were used: 1, 5, 15, 30, 60, 90, 120, 150 and 300 seconds. Tweets were matched by their unique Twitter ID and three dummy variables were generated: one for those that matched, one for those that were only in the streaming API, and one for the tweets that only appear in the search API. For these data, I collected tweets for a period of three minutes on the following six terms: obama, pablo iglesias, داءش (daesh in Arabic), zika, and turtle. Then, to test whether collection time –and, therefore, sample size– is relevant, the same test was performed with one modification: the duration of the collections changed each time to 20, 30, 60, 90, 120, 150 and 300 seconds. The wait time was kept constant constant at 3 minutes and the search terms were the same. These tweets were collected between May 3-6, 2016.

A third test is performed using a third set of data collected on only one term (‘obama’) but with 230 iterations of increasing sample size. A Twarc ‘track’ command in Python collects tweets in real time and runs continuously, and the file created by Twarc is parsed in R at progressively longer intervals in order to include more tweets. I present the analysis of these tests in the results section.

### Data for removal rate analysis

Twitter data were collected on a total of thirty-six topics broken down into three categories: political leaders, important events of political or sociological nature, and trivial terms. In the first category there are nine terms: obama, merkel, hollande, macri, zuma, erdogan, putin, dilma, and rajoy. Other presidents, such as Maduro in Venezuela, were discarded for being too similar to popular words in major languages. The second category, important events, is comprised of fifteen terms: brexit, brussels, capriles, daesh (in English), earthquake, fifa, isis, الدّولة الايسلامية (islamic state in Arabic), mineros, panama, syria, terremoto, turkey, zika, داءش (daesh in Arabic). Lastly, in the category of trivial terms there are twelve different words (both in English and Spanish): cat, gato, koala, lasagna, one, two, panda, perrito, piano, puppy, tortilla, and turtle. These trivial terms were selected such that they would not to generate polarization in online discourse. The total number of groups, thirty-six, was chosen in order to have a critical mass of upper level groups for our multilevel analysis [10]. Tweets were collected from March 16th to April 26th. Within each category, the terms were selected (1) to generate sufficient traffic but not fall within Twitter’s restrictions; (2) author knowledge of the topic; and (3) geographical diversity within each category. The data were collected at different times of the day. Most were collected at different times between 7am and 10pm EST, with a peak between 2 and 3 pm. The time of the day in the country most likely to produce the highest volume of tweets was also considered –for instance, tweets concerning European political figures were collected at different times between 7am and 11pm CET. The peak collection time for these tweets was 8am CET. It is also worth noting that some topics took longer to collect, since they generated lower traffic, while others were faster.

I collected tweets on each of this search terms for a total period of ten minutes in the forward stream per iteration. I then collected them from the search API after a wait time of three minutes, and eliminated these extras and kept the sample within the range indicated by the tweet IDs of the first and last tweet collected in the streaming API. Duplicates were then eliminated and each tweet whose ID matched with a tweet from the other sample was given a 1 and a 0 otherwise. Tweets whose ID appeared in only one of the APIs were given a 1 for being in that sample and a 0 otherwise. The URL for each of these tweets was checked, and if the website returned an error, the tweet was considered to have been deleted. A variable called ‘removed’ was created and a 1 was assigned for tweets that returned an error, and 0 otherwise. This entire process spanned between 20 and 30 minutes per term and iteration, but a tweet was coded in about 15 minutes from the time it was produced until its presence in the website was checked. This is due to the fact that the checking was individual and sequential, with tweets collected first also checked first.

To generate the second dependent variable, removed after 7 days, a slightly different process was used. Given that some terms have relatively large samples of tweets (obama, for instance, has 21,379), and that the process of checking the URL for each tweet is relatively slow (about a second and a half per tweet), tweets were retrieved from the website using the hydrate command from Twarc. This command retrieves the full information from a tweet just from its ID. Hydration produced matches for all but around 5 percent of the sample. To ensure that these tweets had in fact been removed, their URL was checked individually (with their tweet ID) using the same procedure as before. This decreased the total number of removed tweets to about 3 percent on average, which indicates that the hydrate function fails to retrieve the full amount of tweets, even though it does work the vast majority of IDs. Again, removed tweets were given a 1 and those which were still active on Twitter’s site, a 0. Note that I constructed the collection of tweets considering the two aforementioned limitations of the Twitter APIs. On the one hand, tweets that were older than 1 week from the search API could not be downloaded. On the other hand, the collection from the dynamic API was designed such that it would not be affected by Twitter’s restriction of topics that account for more than 1 percent of total traffic. (1) Searches never included more than one term at a time and (2) tweets were not collected when a topic became very popular –for instance, ‘zika’ tweets were collected once the initial wave of attention subsided. A collection was discarded if the likelihood that a topic would produce more that 1 percent of Twitter traffic is high. Only a handful of topics I use in this paper could be subject to rate limiting, and the results hold across all topics under study.

At the tweet level, this process generated independent variables of interest for whether a tweet is in in the search API or in the streaming API. Each of these two variables was coded as 1 if positive and 0 otherwise. The retweet count for every tweet is also in the sample. At the group level, the language of the tweet was included in the dataset and a variable for type (the three categories of terms mentioned above) was created. Other variables of interest in the data are the text of the tweet, the time it was created, and the username of the user who created the tweet. The final sample consists of 205,728 tweets grouped in 36 different terms. Sample size for each term is above 1,000 tweets, with the lowest being ‘zuma’ at 1,036 and the largest ‘obama’ at 21,379. The average sample size for a term is 5,714.6 tweets.

### Statistical analysis

In this section, I briefly explain how the analysis of time was performed and the details of the multilevel model used to analyze removal rates. For the analysis of wait time, I compared how the matching rate of tweets progressed after waiting to collect tweets at the 9 different intervals described above, a process that was repeated 4 times for each term. Linear regression was used to analyze the relationship between wait time and matching rate. I define the ‘matching rate’ as the share of tweets that appear in both APIs. I also analyzed the percentage of tweets, per term, that are *only* in the search API or the streaming API at different wait periods.

To compare how collecting tweets for a longer period of time (which means a larger sample size) increases the matching rate between the two streams, I used the data described above for ‘obama’, which was collected in a loop for 230 iterations at increasing rates of collection time. The data show how the matching rate between the streams evolves with each iteration. Here I call it percent difference, which is the rate of tweets that differ between the streams. Linear regression is used to analyze the relationship between the sample size of each iteration and the matching rate, with the latter as dependent variable.

The modeling technique of choice to analyze removal rates is multilevel analysis. The parameters vary at multiple levels and the data are structured hierarchically, with three different levels nested within each other. The data vary at the tweet level, at the group level –the different topics described above–, and by type of group. For instance, tweet removal or presence in the search or streaming APIs are characteristics specific to the tweets themselves, but variation also exists across different groups as well as different types of groups. Multilevel models recognize this hierarchical structure and allow for residual components at each level in the hierarchy [10–15].

This is important for various reasons. First, if we used a traditional logistic regression, we would need to assume that the observations are independent from each other. If we do not model the hierarchical nature of the data explicitly, we will underestimate the standard errors of the regression and inflate the significance of the results. Secondly, we are interested not just in variation at the tweet level, but in how removal rates vary by group. Third, multilevel models are a better alternative than, for instance, fixed effects in traditional OLS or maximum likelihood models. This is because fixed effects net out the unobservable heterogeneity between groups and produce an estimate that controls for the fixed characteristics of the groups. In multilevel, the random effects estimation allows to model and estimate the heterogeneity between groups, which is better suited to our analysis [12, 16, 17].

This paper begins with a simple random intercepts model that increases in complexity as new covariates and random slopes are added. There are a total of 36 search terms, or 36 *j* categories. For each of these categories, we know the number of individual tweets *n_i_*. Let *y* be a binary response variable of interest. From here, one can build a logistic regression model for the probability *π*_*i*_ of a positive response for each tweet in category *j*:

$$\begin{array}{c} logit\left(\pi_{i}\right)=X_{j}B \end{array}$$ (1)

where X is a matrix of explanatory variables and X_*j*_ is the *j*-th category of X.

The multilevel model allows for partial pooling, which is beneficial when there is variation between groups and when some groups have few observations. Our data are well distributed across groups, so the latter is not a concern in this analysis, but the fact that there is clear variation across groups makes multilevel analysis necessary to correctly estimate the standard errors. To put the modeling of the multilevel set-up more formally:

$$\begin{array}{c} y_{ij}∼N\left(\alpha_{j}+Bx_{ij},\sigma_{y}^{2}\right) \end{array}$$ (2)

$$\begin{array}{c} \alpha_{j}∼N\left(\gamma_{0}+\gamma_{1}z_{j},\sigma_{\alpha }^{2}\right) \end{array}$$ (3)

where B represents the effect at the individual level of covariate *x_ij_*. *α*_*j*_ are the country-specific intercepts, which also follow a normal distribution with variance $\sigma_{\alpha }^{2}$. *γ*_0_ represents the mean of the dependent variable for each group level variable, and *z_j_* is the group level independent variable with its correspondent effect *γ*_1_. This set-up, which may seem relatively complicated, is in fact written in the equation form fairly easily:

$$\begin{array}{c} Y_{i}=X_{i(j)}\beta +Z_{j}\gamma +\mu_{j}+\varepsilon_{i(j)} \end{array}$$ (4)

Here *X* are the set of tweet level covariates, *Z* the group level covariates and *μ*_*j*_ and *ε*_*i*(*j*)_ the error terms for each respectively. *Y* is the response variable, in our case removal rates. This equation represents the more complicated model in this paper with only random intercepts and variables at both the tweet and group levels. Some models are simpler, and only include variables at the tweet level, in which case the terms *Z*_*j*_ *γ* and *μ*_*j*_ would be dropped.

After this brief general introduction to multilevel modeling, I describe the equations used in this article in more detail. We first fit a null or empty two-level model with only an intercept and term effects:

$$\begin{array}{c} log(\frac{\pi_{ij}}{1-\pi_{ij}})=\beta_{0}+\mu_{0j}, \end{array}$$ (5)

where the *log*(*π*_*ij*_/1 − *π*_*ij*_) is the inverse cumulative distribution function of the binomial distribution, also referred to as the logit link function. This paper uses the logit link throughout. The term *β*_0_, the intercept, is shared for all search terms but the random effect *μ*_0*j*_ is specific to each term *j*. As shown above, the random effect is assumed to follow a normal distribution and have variance $\sigma_{u0}^{2}$. This equation will produce the differences in removal rates across terms. Next, an explanatory variable, such as retweet count, is added and the equation changes to

$$\begin{array}{c} log(\frac{\pi_{ij}}{1-\pi_{ij}})=\beta_{0}+\beta_{1}\text{ret}.{\text{count}}_{ij}+\mu_{0j}. \end{array}$$ (6)

Here, the coefficient for retweet count is shared by all search terms –it does not vary. What will change for each search term is its intercept, which will now reflect the inclusion of retweet count as an independent variable —this can be seen in the caterpillar plots in the results section. If between-group variance decreases in the new model, we can conclude that retweet count explains at least some of the differences we observe in removal rates between terms. I will explain some of the surprising changes we observe in our data in the results section.

In the more complex model, we allow both the intercepts and the slopes to vary across groups. We expect that the independent variables in our model will affect our response variable differently depending on the keyword used in the search –we already know that there *is* large variance across groups. Therefore, allowing certain variables to vary across groups will yield a more realistic and meaningful model. We would allow retweet count (the example) to vary by search term by constructing the following formula:

$$\begin{array}{c} log(\frac{\pi_{ij}}{1-\pi_{ij}})=\beta_{0}+\beta_{1}\text{ret}.{\text{count}}_{ij}+\mu_{0j}+\mu_{1j}\text{ret}.{\text{count}}_{ij} \end{array}$$ (7)

Adding a random effect coefficient to the variable retweet count at the group level (*μ*_1*j*_) allows us to obtain a parameter for the independent variable for each group *j*. We can then use this coefficient to understand the effect of retweet count on the removal rate for each group. The tests detailed in the results section include models with multiple independent variables of interest. In those cases, the equation expands to include more variables with beta coefficients if they are not allowed to vary by group, and with both beta and mu coefficients (as in Eq 6) if they are allowed to vary by group to retrieve their random slope.

## Results and discussion

### Results of time analysis

As hypothesized above, there are two mechanisms by which time could affect the samples we obtain from Twitter’s APIs: either there is a lag in updating the search API, which means that the two APIs take some time to converge, or there is a critical point in the sample size collected from the search API at which differences between the two streams plateau. I begin this section by showing that wait time, in fact, does not matter.

**Fig 1:**
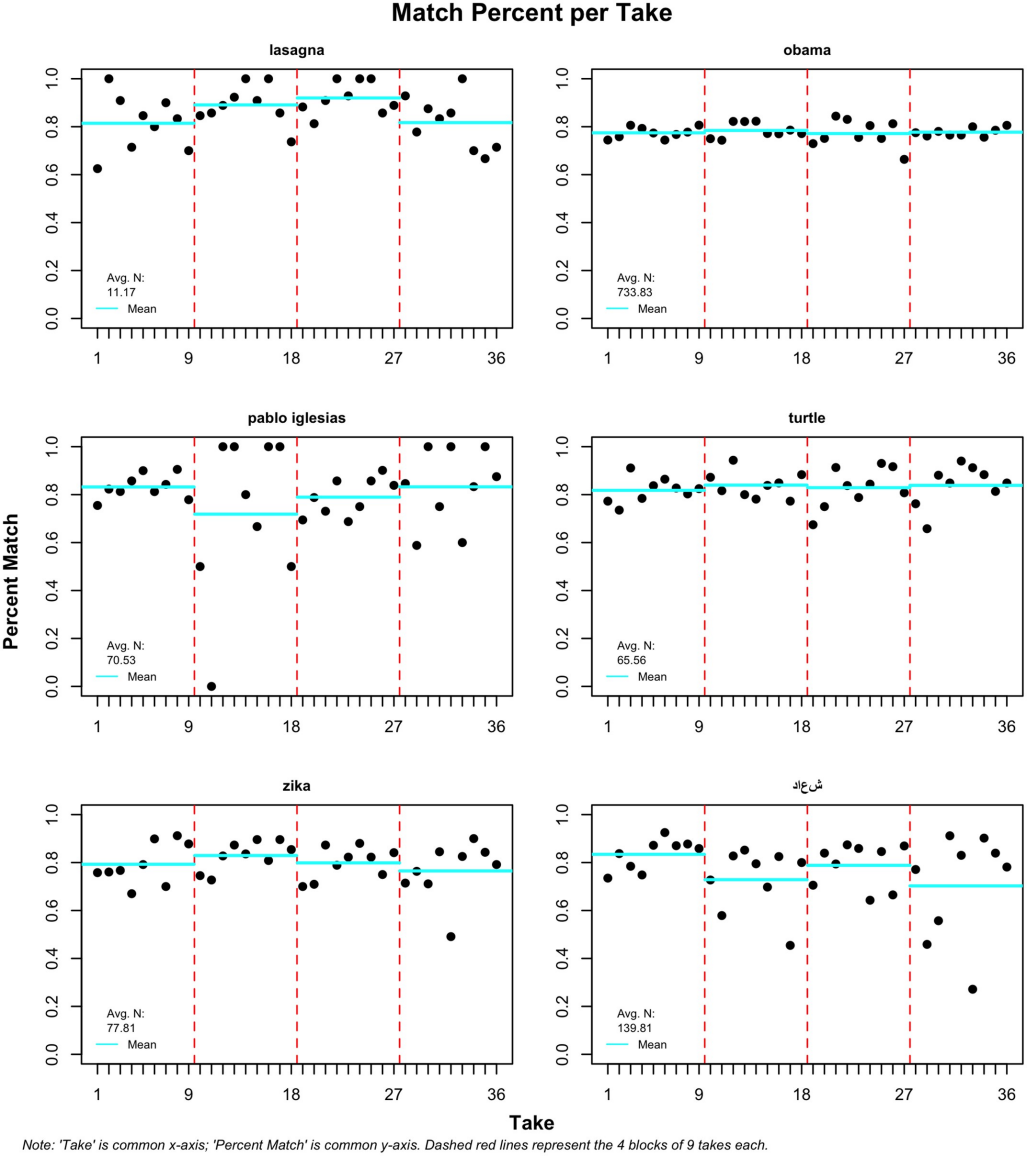
Percent match between streams per topic; four takes of 9 increasing waiting periods.

Fig 1 shows the results obtained after testing six terms, four times, at nine different wait times between streams. The red lines in the plot show the cut points between the four different tests. Within each test, the nine dots show the total matching percentage between the streams at an increasing wait time, from 1 to 300 seconds, as detailed in the data section. These tweets are matched by ID, so we can confidently say that the matching rate is exact as to which tweets appear in both samples. Two important points can be deduced from a simple look at the figure: (1) variability is high for some topics and very low for others, and (2) the dots show no particular positive or negative pattern. The case of ‘obama’ is pertinent: the matching rate remained steadily around 80 percent. There does seem to be a case to be made for the importance of a large N. However, there are undeniable differences between topics that do not obey wait time or sample size, and appear random. Lasagna, for instance, varies rather dramatically between 60 and 100 percent match, with two mean values (blue lines) close to 90 percent. Turtle, on the other hand, never matches perfectly, while ‘pablo iglesias’ matches sometimes but also displays a large amount of variation. ‘Daesh’ also has some cases of low matching rates and means below 80 percent. More needs to be done to understand these differences or otherwise, for some topics, samples may suffer from selection problems.

**Fig 2:**
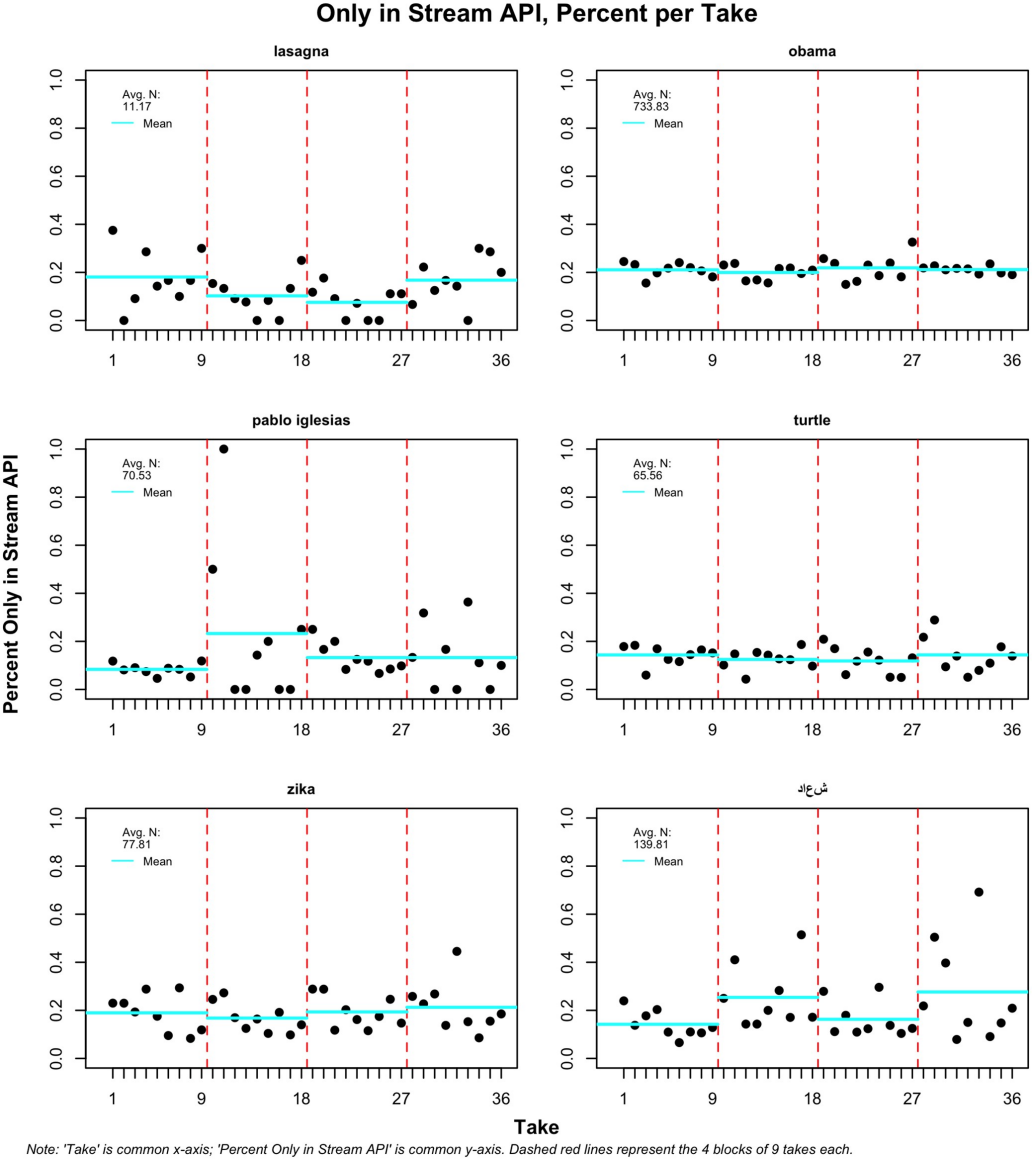
Tweets only in streaming API; four takes per term of 9 increasing waiting periods.

**Fig 3:**
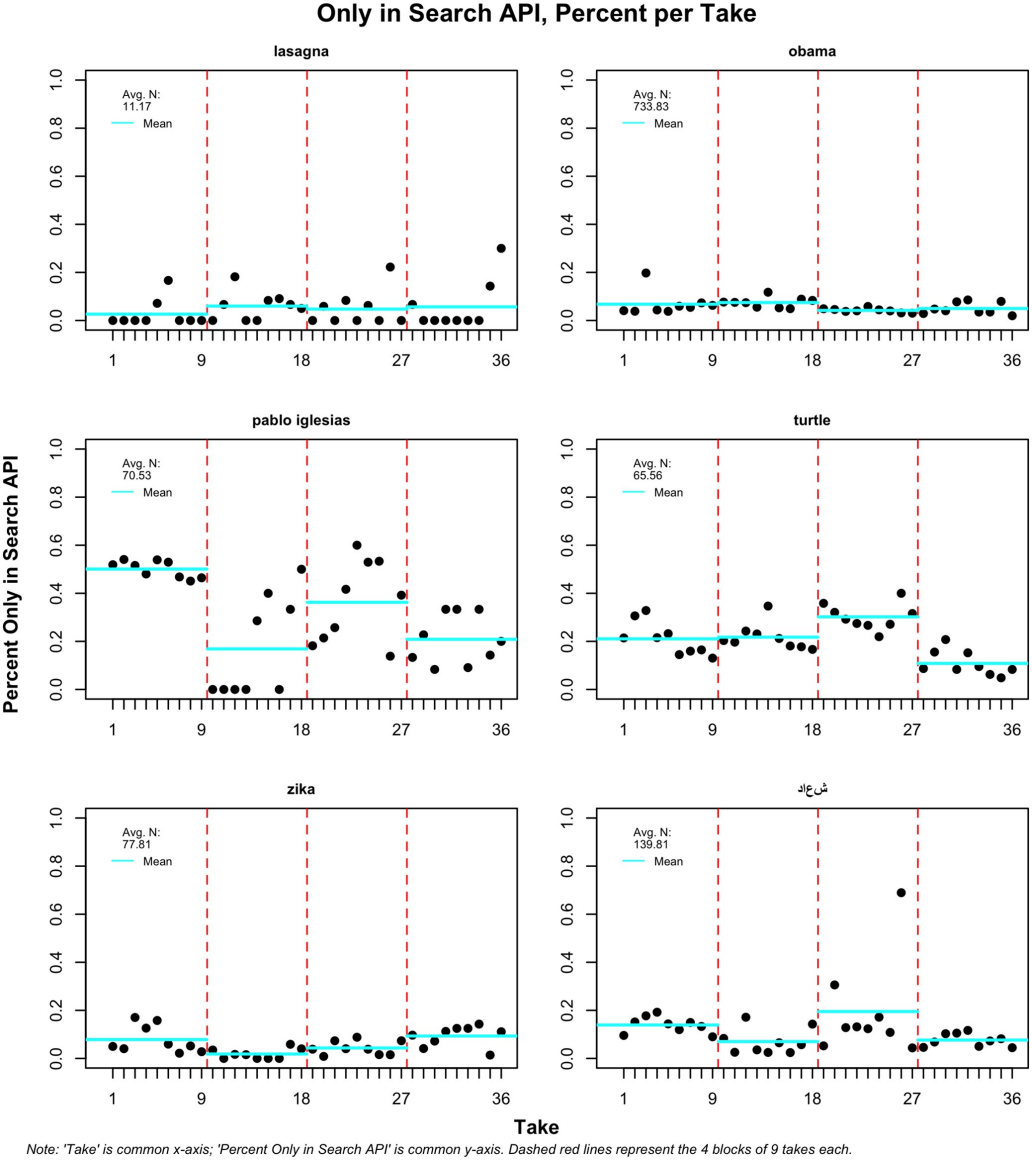
Tweets only in search API; four takes per term of 9 increasing waiting periods.

Another important dynamic to study is whether wait time between collections from both APIs affects the likelihood that a tweet will appear in only one of the two streams. This is important to show as a separate finding because it sheds new light into what so far has been a mystery: do some tweets appear only in the historical API? As Figs 2 and 3 show, this occurs rather often. Fig 2 shows the observations only in the dynamic or streaming API, and Fig 3 shows tweets that appear exclusively in the search or historical API.

In Fig 3, we see that ‘obama’ has a small percentage of observations that is always found only in the historical but not in the dynamic API. It is more striking that terms like ‘pablo iglesias’ or ‘turtle’ have much higher percentages; one test for the former produced a steady 40 percent average. ‘Turtle’ is more consistent at around 20 percent. It is much less surprising that, on average and for most terms, there are more tweets in the streaming API than the search API. ‘Obama’ has, for instance, around 20 percent of tweets that only appear in the dynamic API. This finding is expected, as the search API provides a sample of tweets, which means that some of the ones collected through the dynamic stream will not appear in the historical search.

**Fig 4:**
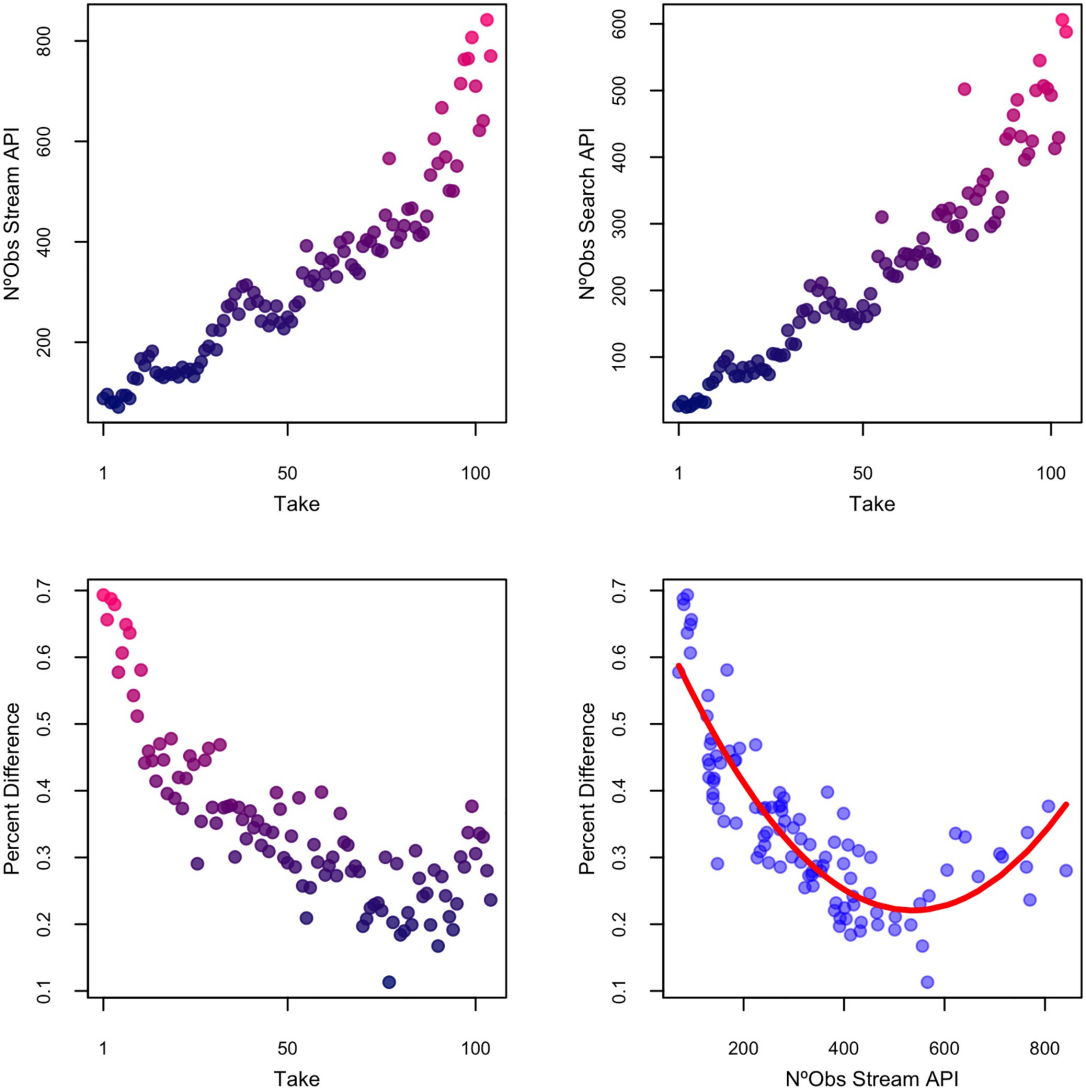
Matching rate for ‘obama’ at increasing sample size and fitted values.

There does not appear to be a systematic lag problem between the two streams, so is it just a question of sample size? Perhaps, after a certain number of tweets, the streams begin to consistently match. The following results point in this direction: the streams converge as more tweets are downloaded, but only up to a point. Above a certain sample size, more tweets no longer guarantee a better matching rate, as Fig 4 shows:

The first two plots in row 1 show that the number of tweets increased monotonically each time we captured new tweets –the ‘take’ number is in the x axis of the first three figures. The leftmost figure in row 2 then shows the percentage of tweets that do not match between each stream at each take. The final figure plots the regression of the number of observations in the forward streaming API (the sample size) on the percentage difference between the streams for each capture of tweets. The results are significant and consistent: At low levels of sample size, the matching rate is small, a percentage that rises sharply as observations increase to about 400. After that, the matching rate plateaus, usually at around 20 to 30 percent and no more convergence occurs beyond this point. The figure shows a u-shaped pattern, but this is due to the fact that there are not many observations and the confidence intervals are wide. As Fig 4 shows, the matching rate of tweets in both APIs becomes flat after about 400 observations.

### Results of removal rate analysis

The analysis of removal rates in our sample yields surprising results with important implications. First, the removal rate of tweets 15 minutes after publication is between 1.75 and 2.5 percent, which is substantial. Unless it is a contentious topic that requires Twitter to take down certain tweets, as we see with the ‘Islamic State’, this range is generally applicable to a wide set of topics. For some issues, however, the natural removal rate of tweets could be above or below this threshold. The researcher should set up similar tests to the ones used in this paper to know whether the topic of interest in more or less likely to have tweets deleted. In Almuhimedi et al.’s [18] sample, only 2.4 percent of tweets are deleted in the period of a *full week*. The authors, however, collected their data based on a random sample of users, not topics. The evidence suggests that, while after a week or so deletions account for about 2.4 percent, removal rates per topic may be higher, mostly if some issues are particularly sensitive.

More strikingly, after a week, the removal rate almost doubled for all our terms: between 3 and 5 percent of tweets in our sample were deleted within a week of publication. This is, indeed, a high rate of removal of tweets and also implies, almost by definition, that there are systematic patterns of removal still unexplored. Certain terms in the dataset, such as ‘Fifa’, have a substantial number of tweets produced by bots, some of which were deleted by Twitter itself. Others, such as those about the ‘Islamic State’, are used for recruitment and also deleted by Twitter. These patterns of content-generation and deletion can shed new light on how organizations, governments, companies, and individuals approach the internet.

A breakdown of removal rates between the three types of terms, i.e. political leaders, political events, and trivial terms, yields even more surprising results. First, while the a priori expectation may be that tweets about political leaders get deleted more often, they are in fact the least removed. In this category, only 1.7 percent of tweets within 15 minutes of publication and 3.16 percent within a week were removed. This stands in contrast to 3.26 percent (15 minutes) and 5.7 percent (1 week) for political events. Trivial terms, which may be expected to suffer the least casualties, in fact had 2.31 percent of tweets removed within 15 minutes and 4.59 percent within a week of publication.

**Fig 5:**
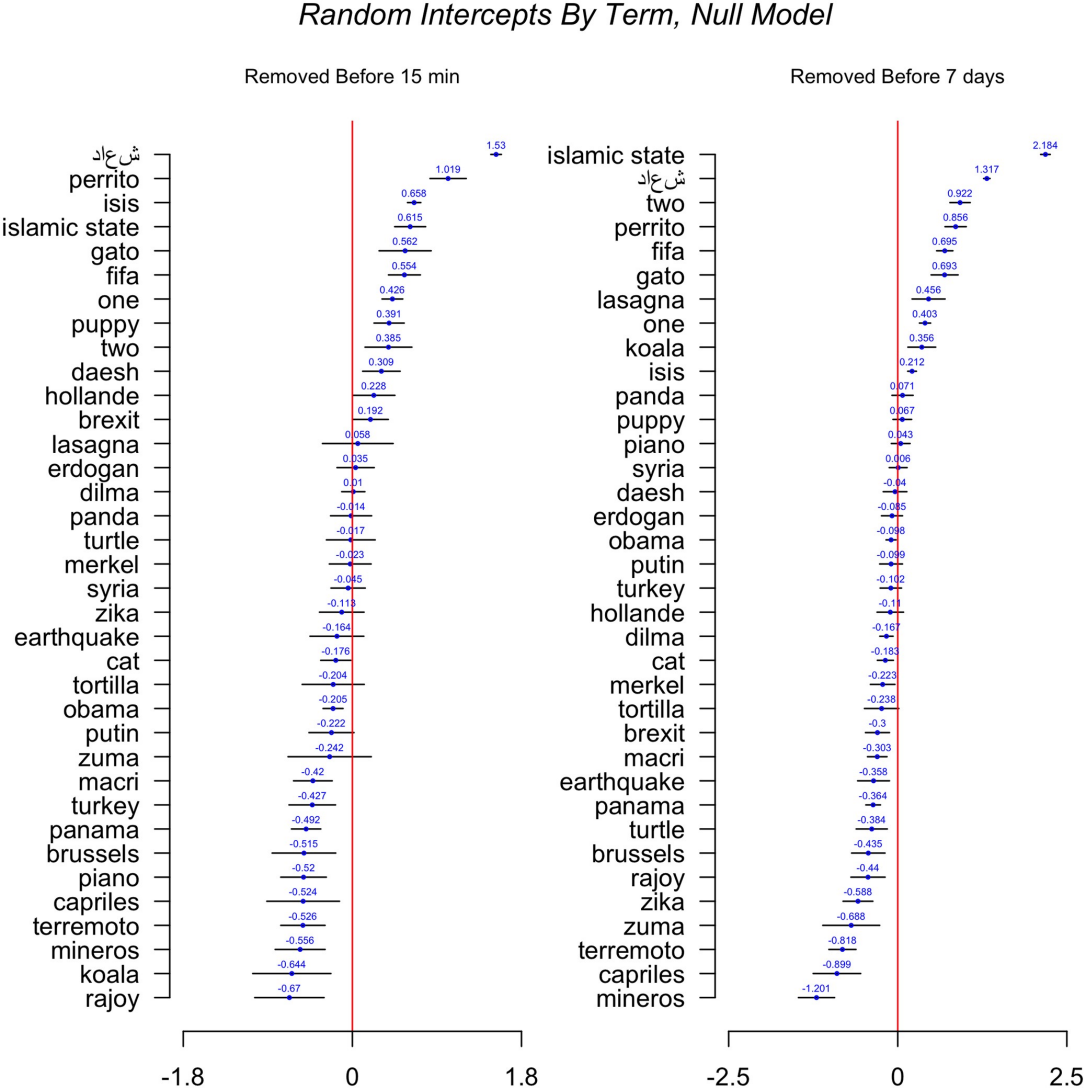
Removal by term, empty model.

Fig 5 shows the results of the first multilevel model. Plotted are the random intercepts per term of the null or empty model.

Twitter’s promise to remove tweets that foster recruitment by the Islamic State appears to have been carried out, as both داءش (‘daesh’) and الدّولة الايسلامية (islamic state) are very much above average in terms of removal of tweets. It is also interesting to note that Twitter appears to take over 15 minutes to remove a lot of these tweets, mostly those that contain the Arabic term for Islamic State. Daesh’s coefficient is similar in both graphs, but the Islamic State’s is much larger on the right side of the graph. Yet ‘Daesh’ is, in fact, a derogatory term in Arabic for the group, and therefore it is unlikely many recruitment tweets are published containing this term. It may surprising to see it being censored so highly, but upon closer inspection, we see that it is hard to distinguish them from pro-Islamic State propaganda. Twitter’s algorithms must punish users who tweet about ‘daesh’ thinking that they are pro-Islamic State. In fact, it is surprising that الدّولة الايسلامية is removed at the same rate as ‘isis’, its equivalent in latin alphabet. After 7 days, we see that they diverge substantially, with the latter barely above zero and the former gaining almost outlier status at the top of the chart.

The other terms also show surprising patterns. Some terms, such as ‘fifa’, show higher levels of removal rates. Closer analysis of these tweets shows that the deletions are mostly driven by bots. During the crisis of the Panama papers, in which a few top-ranking Fifa officials were implicated, bots appeared to send out positive information about the organization in an effort to promote positive hashtags. This is clear evidence that a tactic initially developed by marketing companies is now being used by organizations –and potentially governments– to prop up reputations in times of crises. The data show that some of these bots are removed by Twitter, but not all.

Another interesting takeaway is that there is a stochastic component to the removal rates of a lot of the topics –‘gato’ is removed much more often than ‘isis’, for instance, and ‘piano’ is right in the average while charged topics such as ‘brexit’, ‘panama’ or ‘zuma’ are below-average. ‘Perrito’, the Spanish equivalent of ‘puppy’, is deleted at a much higher rate than ‘islamic state’ in Arabic before 15 minutes. Lastly, it is important to take note of the two terms that appear significantly below average in the rightmost graph: ‘capriles’ and ‘mineros’. These two terms refer to the well-known Venezuelan opposition leader and to a crisis that took place in March, 2016 in Venezuela, in which a grave with the bodies of 26 miners was found and a crisis for the government started. While no exact explanation can be provided at this point, this anomaly in the data suggests that an unknown factor is helping these tweets survive at much higher rates than others. Perhaps an organized campaign against the regime, which would generate a lower number of deletions if well-planned, or an unusual number of retweets with none of the original tweets being deleted, could be the cause.

**Fig 6:**
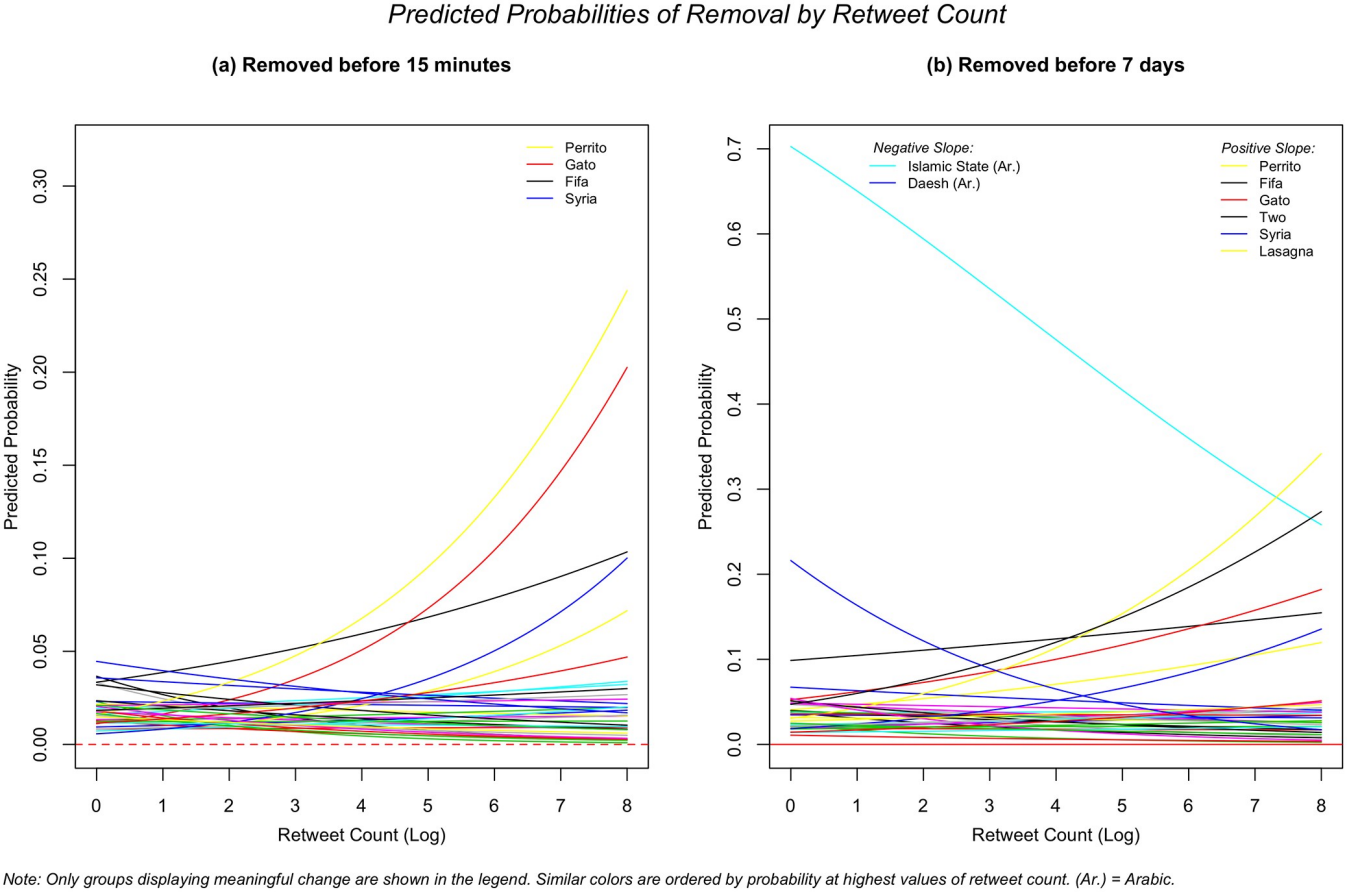
Predicted probabilities of removal by retweet count.

In Fig 6, I test whether the total retweet count of a tweet increases its chances of being removed. The logic of the hypothesis is as follows: the more popular a tweet gets, the more likely it is to be censored either by an overzealous regime or the user him/herself. The results show little evidence that this is the case. Fig 6a and 6b show the predicted probabilities from the random slope model for the effect of the retweet count (logged) on the removal of a tweet 15 minutes and one week after publication. The graphs show the lack of significance of the models, both in the fixed effects and the variance between groups. A few terms, such as the Islamic State, show particular patterns, but in the case of ‘gato’ or ‘perrito’ this is more likely due to the lack of observations as observed in the density plot. Overall, there is no statistically significant difference between groups or in the fixed effects coefficient for retweet count. The predicted probabilities show that, for some terms, the effect is significant (‘daesh’ and ‘islamic state’ in Fig 6b), but the overall pattern is inconclusive. With a large sample of terms we could better establish whether these cases are mere outliers or indicators of substantial patterns —the legend only shows the names of the groups that display a different pattern from the majority of groups.

**Fig 7:**
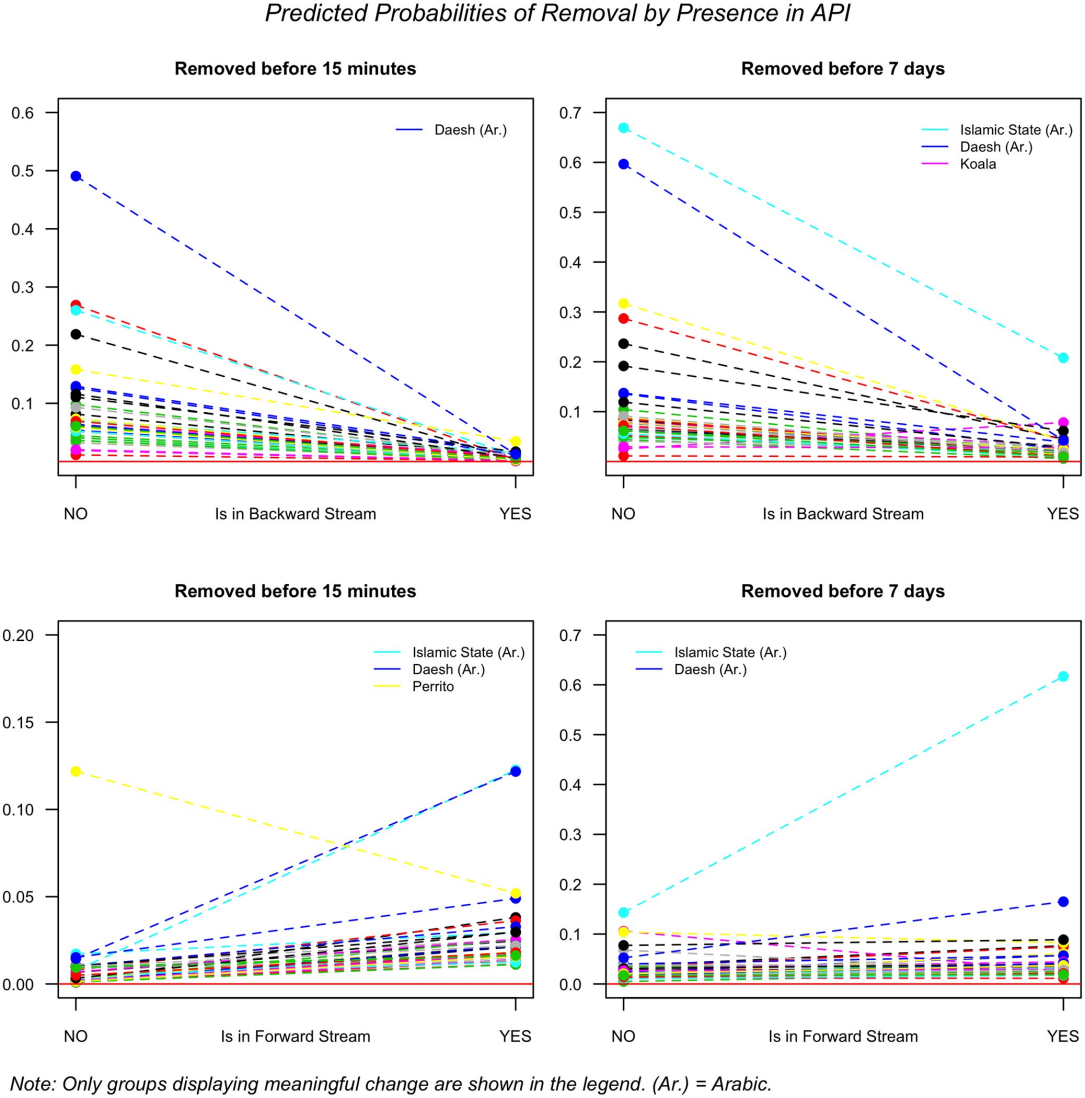
Predicted probabilities of removal by presence in API.

A more surprising result is presented in Fig 7, which shows the the predicted probabilities of a tweet being removed if it is in the search API (backward) or the streaming API (forward). We can see that within 15 minutes of publication, all terms follow similar patterns of removal rate in both streams (left figures). We observe that the probability that a tweet is deleted decreases if it appears in the search API. Fig 7 displays the predicted probability of a tweet being removed as a function of whether it appears in the search or the dynamic API. The dashed lines illustrate the change that some terms experience but do not reflect predictions, since the variable is dichotomous. This is interesting because tweets that make it into the search API are usually more relevant tweets. Twitter itself selects these tweets according to their own algorithm, and not just as a function of the retweet count. Conversely, tweets that appear in the dynamic API are much more likely to be deleted across all terms.

The plots on the right side of the figure show that, for tweets removed within a week, there is a small loss of consistency between the terms. In the backward stream (top right), some terms have a much sharper negative slope, such as داءش (daesh) and الدّولة الايسلامية (islamic state), while one has a positive slope (koala). In the forward stream, differences are starker. Most terms experience a marked decrease, and a few more have a flat or negative slope. Only the islamic state maintains a positive slope, as does daesh, but both are less pronounced.

Table 1 reports the results of the four models from Fig 7. The fixed effects terms in Models 1 and 2 show that there is a strong and negative relationship between a tweet being in the search API and the the likelihood that it will be removed. The random effects terms indicate that the variance between groups is not significant. In Fig 7, it appeared that some terms were visually different from each other, but the model shows that these differences are not significant –and they remain significant if we run the models without the apparent outliers. The fixed effects terms in Models 3 and 4, on the other hand, show a very strong positive association between the forward streaming API and a tweet being deleted across terms. It may appear in Fig 7 that the lines are flat, but the predicted probabilities at 0 and at 1 show clear positive patterns in the bottom graphs of the figure. The results hold if we run the tests without the outliers in the sample, which corroborates the overall pattern. Yet again, the random effects coefficients in Models 3 and 4 evince the lack of statistically significant differences among terms.

**Table 1:** Fixed effects coefficients and random effects variance for removal rates per stream.

	Model 1 *15 min*		Model 2 *7 days*		Model 3 *15 min*		Model 4 *7 days*	
	Fixed Eff.	Random Eff.	Fixed Eff.	Random Eff.	Fixed Eff.	Random Eff.	Fixed Eff.	Random Eff.
In Search API	-1.421*** (0.116)	0.638 (0.799)	-2.846*** (0.157)	0.841 (0.917)				
In Stream API					0.464*** (0.122)	0.541 (0.736)	1.863*** (0.195)	1.385 (1.177)
Intercept	-2.326*** (0.137)	1.002 (1.001)	-2.596*** (0.138)	0.760 (0.872)	-3.642*** (0.133)	0.632 (0.795)	-5.606*** (0.203)	1.779 (1.334)
Observations	205,728		205,728		205,728		205,728	
Log Likelihood	-33,006.420		-18,913.040		-35,866.910		-22,854.860	

*** *p* = 0.001,

** *p* = 0.01,

* *p* = 0.05

## Conclusions

These results yield a few important takeaways, all of which provide broad new avenues for research. First, there does not appear to be much of a lag between the stream and search APIs, but the number of tweets common in collections from both APIs will depend on sample size. This relates to an important parallel finding, namely, that above 400 tweets the matching rate between the streams plateaus and most terms will permanently have between 20 and 30 percent of tweets that do not appear in the search API. This has obvious implications for sample reliability and analysis of networks. Second, there is a number of tweets that only appears in the search API, contrary to what was expected. The proportion varies slightly between terms, but for terms like ‘obama’ or ‘zika, which have larger sample sizes, it ranges between 3 and 6 percent. This finding is important to consider when collecting samples only from the streaming API. It is best for the researcher, if possible, to set up a system in which both APIs collect data, removing duplicates afterward. This would ensure that the sample includes the most representative sample of tweets available outside Twitter’s firehose.

Third, I find that tweets get removed at a high rate –between 2 and 2.5 percent within 15 minutes of publication and 3 to 5 percent within a week. There is also evidence that tweets about presidents are removed less often than tweets on politically charged issues, and also less often than tweets about trivial topics. Indeed, it is expected that politically charged topics are removed in greater numbers, but it is less intuitive that the same should happen with non-contentious topics such as food or domestic animals. Indeed, an important research agenda is to explore the mechanisms by which individuals self-regulate or censor their own content. This article has not dealt with this issue explicitly, but it is indeed crucial to understand how it occurs. The results introduced here should provide certain puzzles (for instance, why do certain trivial categories experience greater rates of removal than some politically charged topics?) and some direction for future research. Lastly, tweets tend to be removed more often if they are in the streaming API, which responds to the fact that it includes a very large majority of all published tweets. The fact that the difference between the streams persists after 15 minutes and also exists within 1 week of publication also reflects the extent to which the search API is filtered by Twitter to include more relevant tweets, which affects the type of sample one collects through the search API.

There are at least two ways in which this paper can be expanded. First, more data should be gathered on a wider variety of topics, which would increase our upper level variation and produce more complete and interesting results. 36 groups is not a small sample, and thus the results in this paper should not be affected by sample size [10]. For the nature of our study, however, it would be fitting to obtain data for a wider range of groups, which could reveal greater differences in upper level terms. We could then build models that explained inter-group variation in our removal rate analysis.

Second, while this paper begins to explain in detail some aspects of Twitter’s search and streaming API that have so far remained a black box, more needs to be done in this regard. Researchers are increasingly interested in the use of Twitter data, and rightly so, for its abundance and the new possibilities it affords. Yet, knowledge on how the streams work is still incomplete. This article seeks to fill this gap by showing new dynamics within Twitter’s APIs and offering a new approach to the study of removal rates of tweets.

## Supporting information

S1 Table This document contains descriptive statistics. (PDF)
