# Supplementary material for: Where in the world is my tweet: Detecting irregular removal patterns on Twitter
Source: PLoS One. 2018 Sep 20;13(9):e0203104. doi: 10.1371/journal.pone.0203104 (PMC6157815; doi:10.1371/journal.pone.0203104)
Supplement: S1 Table — (PDF) [file pone.0203104.s001.pdf]

Supporting information

S1 Table: Desciptive Statistics

Statistic	N	Mean	St. Dev.	Min	Max
Removed (15 minutes)	205,728	0.026	0.159	0	1
Removed (1 week)	205,728	0.047	0.212	0	1
Retweet count all (log)	204,634	1.806	2.304	0.000	12.129
Present in Search API	205,728	0.765	0.424	0	1
Present in Stream API	205,728	0.789	0.408	0	1
